# The effectiveness of 4DCT in children and adults: A pooled analysis

**DOI:** 10.1002/acm2.12488

**Published:** 2018-11-09

**Authors:** Sophie C. Huijskens, Irma W. E. M. van Dijk, Jorrit Visser, Brian V. Balgobind, Coen R. N. Rasch, Tanja Alderliesten, Arjan Bel

**Affiliations:** ^1^ Department of Radiation Oncology Amsterdam University Medical Center University of Amsterdam Amsterdam The Netherlands

**Keywords:** 4DCT, pediatric RT, respiratory‐induced motion

## Abstract

**Background:**

While four‐dimensional computed tomography (4DCT) is extensively used in adults, reluctance remains to use 4DCT in children. Day‐to‐day (interfractional) variability and irregular respiration (intrafractional variability) have shown to be limiting factors of 4DCT effectiveness in adults. In order to evaluate 4DCT applicability in children, the purpose of this study is to quantify inter‐ and intrafractional variability of respiratory motion in children and adults. The pooled analysis enables a solid comparison to reveal if 4DCT application for planning purposes in children could be valid.

**Methods/materials:**

We retrospectively included 90 patients (45 children and 45 adults), for whom the diaphragm was visible on abdominal/thoracic free‐breathing cone beam CTs (480 pediatric, 524 adult CBCTs). For each CBCT, the cranial–caudal position of end‐exhale and end‐inhale positions of the right diaphragm dome were manually selected in the projection images. The difference in position between both phases defines the amplitude. Cycle time equaled inspiratory plus expiratory time. We analyzed the variability of the inter‐ and intrafractional respiratory‐induced diaphragm motion.

**Results:**

Ranges of respiratory motion characteristics were large in both children and adults (amplitude: 4–17 vs 5–24 mm, cycle time 2.1–3.9 vs 2.7–6.5 s). The mean amplitude was slightly smaller in children than in adults (10.7 vs 12.3 mm; *P* = 0.06). Interfractional amplitude variability was statistically significantly smaller in children than in adults (1.4 vs 2.2 mm; *P* = 0.00). Mean cycle time was statistically significantly shorter in children (2.9 vs 3.6 s; *P* = 0.00). Additionally, intrafractional cycle time variability was statistically significantly smaller in children (0.5 vs 0.7 s; *P* = 0.00).

**Conclusions:**

Overall variability is smaller in children than in adults, indicating that respiratory motion is more regular in children than in adults. This implies that a single pretreatment 4DCT could be a good representation of daily respiratory motion in children and will be at least equally beneficial for planning purposes as it is in adults.

## 1 Introduction

Precise knowledge of organ motion is extremely important for high‐precision image‐guided radiotherapy, aiming for an optimal balance between accurate target coverage and minimizing dose to surrounding healthy tissues. As the field of radiotherapy is expanding rapidly, with proton and carbon ion therapies, the need for high accuracy is of increasing importance.^1^ This holds especially in pediatric radiotherapy, where dose to healthy surrounding tissues is associated with a highly unfavorable increased risk of developing adverse events later in life.^2^ Particularly, respiratory‐induced organ motion is one of the main challenges to deal with during abdominal radiotherapy. Continuous developments and research have focused on accounting for respiratory‐induced organ motion in radiotherapy.^3^, ^4^, ^5^

Typically, safety margins surrounding the tumor and organs at risk are determined to account for inter‐ and intrafractional organ motion, setup variations, and delineation errors.^6^, ^7^, ^8^ In adults, pretreatment four‐dimensional computed tomography (4DCT) is often acquired to quantify respiratory‐induced organ motion in order to assess the intrafractional component of the safety margin. With the 4DCT technique, the image acquisition is related to the patient's respiration and is binned in a number of uniform respiratory phases.^9^ This results in a series of reconstructed 3DCT scans representing the entire respiratory cycle, thereby encompassing the full range of respiratory‐induced organ motion. However, day‐to‐day (interfractional) variability and irregular respiration (i.e., intrafractional variability) have shown to be limiting factors of the 4DCT technique and application for treatment planning in adults.^10^, ^11^ First of all, 4DCT acquisition captures a single time‐point while there might be variability of respiratory motion during different treatment days. Adult studies have investigated the predictive value of a single 4DCT for a variety of treatment sites and have reported both positive and negative on it.^12^, ^13^, ^14^, ^15^ Besides, the 4DCT images are often subject to motion artifacts mostly resulting from irregular respiration, which causes misidentification of the respiratory cycles. Although these limitations are present, 4DCT provides useful information for planning purposes and is routinely applied for highly mobile tumors in adults. However, in pediatric radiotherapy a 4DCT is not commonly applied, since the 4DCT acquisition requires extra patient training and treatment time. Additionally, a 4DCT involves a slightly higher imaging dose and due to the ALARA (keeping doses As Low As Reasonable Achievable) principle, reluctance remains to use 4DCT in the pediatric population. Nevertheless, for mobile targets in the thoracic and abdominal region, such as neuroblastomas, Wilms’ tumors and lung metastases, imaging with 4DCT might yield a more precise treatment. This lowers the risk of adverse effects, but the additional imaging should be weighed against increased imaging dose to the patient.

We recently quantified respiratory‐induced diaphragm motion, as a surrogate for motion of upper abdominal and thoracic target volumes and organs at risk, in 45 children^16^ and concluded that this respiratory‐induced diaphragm motion was smaller and more regular in children than previously reported by another group in adults,^17^ indicating that a pretreatment 4DCT could also be promising in pediatric radiotherapy. However, respiratory‐induced diaphragm motion in adults was quantified using a partly different methodology in lung cancer patients.^17^ Since these patients suffered from lung pathologies, it is likely that respiration was affected and comparison with our pediatric data^16^ was inconclusive. A solid comparison of respiratory characteristics in children and adults that could affect 4DCT image quality and its effectiveness requires the same clinical image‐guided practice, analysis and similar tumor sites, that is, excluding adults with lung tumors.

Therefore, in this study we aimed to quantify the inter‐ and intrafractional variability of respiratory‐induced diaphragm motion in adults, using the exact same methodology as used in our pediatric study.^16^ The pooled analysis of pediatric and adult data enables a methodologically consistent comparison of respiratory motion characteristics in children and adults in order to reveal if 4DCT application for planning purposes in children could be valid.

## 2 Materials and methods

### 2.A Patient data

Pediatric data was available from our previous study, where respiratory‐induced diaphragm motion was retrospectively analyzed during the treatment course of 45 children (median age 11; range 2–18 yr).^16^ We collected information on general anesthesia (GA, *n* = 7), and patient characteristics including age at the first radiation treatment fraction, height, weight, primary cancer diagnosis, and radiation site. A detailed overview of pediatric patient characteristics is given in Huijskens et al.^16^ and added in Supporting Information Table S1. For this pooled analysis, 45 adults (median age 63; range 34–93 yr), treated at our institute within the same period (2010–2016) as the pediatric group, were randomly included when the diaphragm was visible in upper abdominal or thoracic free‐breathing cone beam computed tomography (CBCT) scans. To prevent bias when comparing the respiration pattern in the adult group to that of the pediatric group, lung cancer patients were excluded. Thus, the selection yielded esophageal (*n* = 13), gastric (*n* = 17), and pancreatic (*n* = 15) cancer patients. Supporting Information Table S2 provides a detailed overview of the adult patient characteristics. In our institute, abdominal compression to control respiratory motion is neither used in children, nor in adults. A general overview of all patient characteristics can be found in Table 1.

**Table 1:** Characteristics of included patients

	Children	*N* = *45* (%)	Adults	*N* = *45* (%)
Gender				
Male	28	(62)	31	(69)
Female	17	(38)	14	(31)
Age at first RT fraction (yr)				
Mean (median; range)	11 (11; 2–18)		61 (63; 34–93)	
0–5	6	(13)		
6–10	12	(27)		
11–18	27	(60)		
30–49			9	(20)
50–69			26	(58)
≥70			10	(22)
Height (cm)				
Mean (median; range)	144 (148; 90–186)		175 (175; 134–203)	
Weight (kg)				
Mean (median; range)	40 (37; 15–81)		71 (69; 52–134)	
Type of primary cancer				
CNS tumor^a^	20	(44)		
Sarcoma^b^	16	(36)		
Neuroblastoma	4	(9)		
Renal tumor^c^	2	(4)		
Other^d^	3	(7)		
Esophagus			13	(29)
Pancreas			15	(33)
Stomach			17	(38)
Radiation site				
(Cranio)spinal	20	(44)		
Thoracic/mediastinal	18	(40)		
Abdominal (incl. flank)	7	(16)		
Upper abdominal			45	(100)
Total number of CBCTs	480		524	
Mean (median; range)	11 (7; 4–32)		12 (11; 2–30)	
Rotation (degrees)				
200	35	(78)		
360	10	(22)	45	(100)
Acquisition parameters				
120 kV, 10 mA, 10/40 ms	45	(100)	45	(100)

a Including: anaplastic glioma (*n* = 1), ependymoma (*n* = 1), germinoma pinealis (*n* = 4), medulloblastoma (*n* = 14).

b Including: Ewing sarcoma (*n* = 10), rhabdomyosarcoma (*n* = 5), osteosarcoma (*n* = 1).

c Wilms’ tumor (*n* = 1), clear cell carcinoma (*n* = 1).

d Including: lymphoma (*n* = 2), desmoplastic small round cell tumor (*n* = 1).

RT: Radiotherapy; CNS: central nervous system; CBCT: cone beam computed tomography.

### 2.B CBCT acquisition

In our pediatric study, a total of 480 pediatric CBCTs (median 7; range 4–32 per patient) were included.^16^ Acquisition parameters for pediatric CBCTs were 120 kV, 10 mA, and 10 or 40 ms exposure time per projection. The rotation varied from 200 (*n* = 35) to 360 (*n* = 10) degrees, resulting in a variation in number of projection images per CBCT (180–760). Adult patients received daily or weekly CBCT imaging (Synergy, Elekta Oncology systems, Crawly, UK) prior to treatment for position verification, totaling 524 CBCTs (median 11; range 2–30 per patient). Acquisition parameters for adult CBCTs were 120 kV, 10 mA, and 10 or 40 ms exposure time per projection. For all adults, the rotation yielded 360 degrees, resulting in approximately 760 projection images per CBCT.

### 2.C Diaphragm tracking

Identical to the methodology used for the pediatric group^16^ an adapted version of the Amsterdam Shroud (AS) method^17^, ^18^ was used to track diaphragm motion from CBCT imaging. For each CBCT, a two‐dimensional AS image was created. Along the horizontal axis of this image, representing the projection images, we manually selected the projection images corresponding to end‐exhale and end‐inhale positions of the diaphragm. In each of those selected projection images, we then manually determined the cranial–caudal (CC) position of the top of the diaphragm (i.e., corresponding to the peak‐to‐peak position variation). Subsequently, the pixel coordinate corresponding to the position of the top of the diaphragm was translated to millimeters relative to the patients’ planned isocenter, by including a magnification correction to account for the difference in scale between the imaging panel and the isocenter.^19^ Additionally, we corrected for the geometry of the CBCT scanner.^19^ This resulted in a respiratory pattern describing the CC position of the diaphragm in end‐exhale and end‐inhale phases over the course of CBCT acquisition (detailed overview shown in Supporting Information Figure S1).

### 2.D Respiratory analysis

The amplitude was defined as the difference in CC position of the diaphragm between end‐exhale and end‐inhale phases. The cycle time described the time between two consecutive end‐inhale positions. Day‐to‐day variation was expressed as interfractional variability (i.e., the SD over mean amplitudes from each fraction), and irregular breathing was expressed as intrafractional variability (root mean square of the SDs from each fraction).

For the adult patients, we calculated the same parameters as in our pediatric study; mean amplitude, interfractional variability, and intrafractional variability (see schematic overview; Supporting Information Figure S1). For the whole patient group, including both children and adults, we calculated the group mean amplitude by averaging the patients’ mean amplitude, the group interfractional variability by averaging the patients’ interfractional variabilities, and the group intrafractional variability by averaging the patients’ intrafractional variabilities. Calculations of these respiratory parameters were also computed for the cycle time.

### 2.E Statistical analysis

Since not all data fitted the normal distribution (tested with the Shapiro–Wilks test), differences in mean amplitude, mean cycle time, and inter‐ and intrafractional variabilities in children and adults were tested for significance with the Mann–Whitney *U* test, considering *P* < 0.05 significant. This comparison also provides insight into possible explanations on respiratory‐induced motion based on continuous values of age, height, and weight. Therefore, we used the Spearman's correlation test (significance level *P* < 0.05) to test for possible relationships between respiratory‐induced diaphragm motion parameters and patient‐specific factors (age, height, and weight). For the pediatric group separately, we tested with the Mann–Whitney *U*‐test (significance level *P *< 0.05) whether respiratory parameters of children treated under GA (*n* = 7, age range 2–11 yr) differed from children treated without GA in a similar age range (*n* = 12, age range 3–10 yr).

## 3 Results

**Figure 1:**
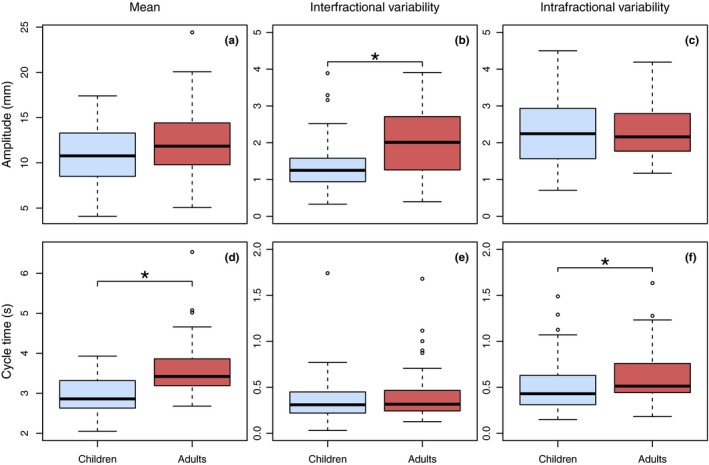
Boxplots showing the distributions of the individual means and standard deviations of the amplitude (upper row, a, b, and c) and cycle time (bottom row, d, e, and f) of respiratory‐induced diaphragm motion in children (blue) and adults (red). Boxes: median value and upper and lower quartiles; whiskers: lowest and highest data point within 1.5 × interquartile range; circles: outliers. (Color figure online only). Note: y‐axes differ in range. * significant differences (*P* < *0.05*)

**Figure 2:**
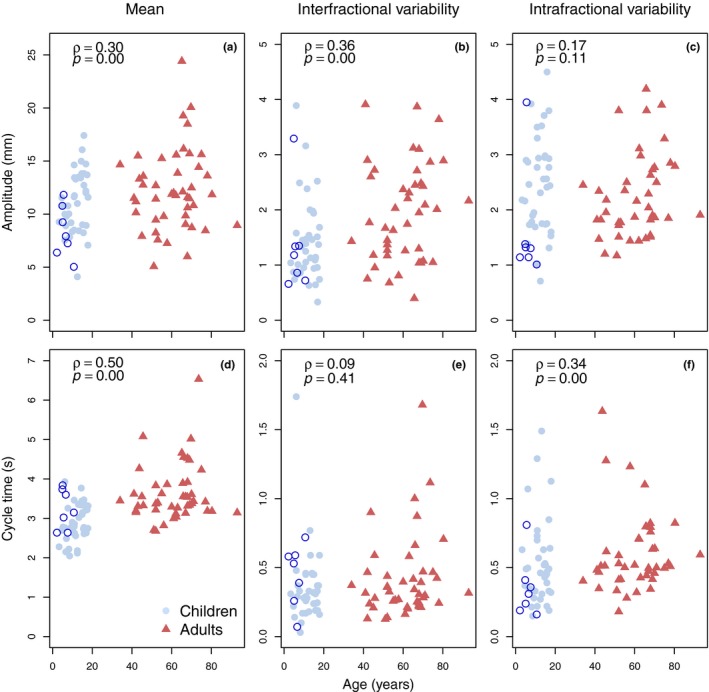
Scatter plots describing relations (Spearman's *ρ* and *P*‐value) between the amplitude (upper row, a, b, and c) and cycle time (bottom row, d, e, and f) of respiratory‐induced diaphragm motion and age (significance level: *P* < *0.05*). Dots (light blue), open circles (dark blue) and triangles (red) represent respectively pediatric patients treated without and with anesthesia, and adult patients. (Color figure online only). Note: y‐axes differ in range.

The differences in respiratory‐induced diaphragmatic motion parameters between children and adults are summarized in Fig. 1. The mean amplitude was slightly smaller in children than in adults (average: 10.7 vs 12.3 mm, range: 4.1–17.4 mm vs 5.1–24.4 mm), however, statistically insignificant (*P* = 0.06). Interfractional amplitude variability was statistically significantly smaller in children than in adults (average: 1.4 vs 2.2 mm, range: 0.3–3.9 vs 0.4‐7.2 mm; *P* = 0.00). Mean cycle time was statistically significantly shorter in children (average: 2.9 vs 3.6 s, range: 2.1–3.9 vs 2.7–6.5 s; *P* = 0.00), since children breath faster than adults. Additionally, intrafractional cycle time variability was statistically significantly smaller in children (0.5 vs 0.7 s, range: 0.2–1.5 vs 0.2–3.2 s; *P* = 0.00). The intrafractional amplitude variability was significantly smaller in children treated under GA (1.6 mm) than in children of similar ages treated without GA (2.4 mm), other respiratory‐induced diaphragm motion characteristics did not differ (Fig. 2). The repeated analysis, with exclusion of the children treated under GA, did not change our results, when comparing children and adults.

Possible relationships between respiratory‐induced diaphragm motion parameters and patient‐specific factors, over continuous values of age, height, and weight, are given in Fig. 2 and Supporting Information Figure S2. All correlations of mean amplitude and mean cycle time with age, height, and weight were statistically significant (*P *< 0.05). However, values of Spearman's correlation coefficients were low (amplitude *ρ *≤ 0.30; cycle time *ρ *≤ 0.50). Correlations of inter‐ and intrafractional amplitude and cycle time variabilities with patient‐specific factors were all statistically insignificant, except for interfractional amplitude variability with age (*P *= 0.00; *ρ *= 0.36) and weight (*P *= 0.04; *ρ *= 0.22), and intrafractional cycle time variability and age (*P *= 0.00; *ρ *= 0.34).

## 4 Discussion

Respiratory motion characteristics of 90 patients, including 45 children and 45 adults, analyzed with identical methodology in 1004 CBCTs,^16^ were compared to reveal the effectiveness of 4DCT application for planning purposes in pediatric radiotherapy. This comprehensive dataset shows small but statistically significant differences in respiratory‐induced diaphragm motion in children and adults. We found that respiratory motion in children during the treatment course is more regular, indicating that a 4DCT will be at least equally beneficial for planning purposes as it is in adults. Additionally, large ranges of mean amplitude and mean cycle time in both children and adults confirm that respiratory motion is patient‐specific and requires an individualized approach (e.g., based on 4DCT) to account for. This was emphasized by weak correlations between all respiratory parameters and the patient‐specific factors.

Unexpectedly, we found that interfractional variability of the amplitude was statistically significantly smaller in children than in adults, meaning that over all fractions the respiratory amplitude was more stable in children. This could be explained by the fact that, since it is known that patients experience radiotherapy as a stressful procedure,^20^ more attention in the clinic is paid toward comforting the child and reducing anxiety,^21^, ^22^, ^23^ while this is less introduced in the clinic for adults. This might have also led to a more constant cycle time during each fraction in children than in adults, as shown by the smaller intrafractional variability of cycle time in children.

These present results indicate that a single pretreatment measurement of respiratory‐induced motion with 4DCT could be a good representation for motion in children during radiotherapy. Moreover, large variation in amplitude and cycle time in both children and adults confirms that a more individualized approach with 4DCT can be effective for children as well. Recently, discussion is ongoing on radiation risks in children from medical imaging.^24^, ^25^, ^26^, ^27^, ^28^ However, although 4DCT increases imaging dose 2–4 times compared to 3DCT,^29^, ^30^ it provides more detailed information on organ motion, leading to more precise treatment planning and potentially minimizing dose to healthy tissues. Recently, for this aim a pediatric‐specific 4DCT scanning sequence and protocol was developed.^31^ In our institute, we recently introduced 4DCT for children and applied the same 4DCT protocol as used for adults. However, when feasible, parameters were adjusted to achieve lower imaging doses. Similarly, in our institute, a low‐dose protocol for CBCT imaging was developed and implemented for pediatric patients.^32^ For all adults, CBCT imaging was acquired with 360 degrees rotation while pediatric CBCTs were acquired at lower imaging doses with 200–360 degrees rotations. This resulted in a variation in number of projection images (180–760) between children and adults. However, for each patient, a sufficient amount of projection images was available for tracking diaphragm motion, representing sufficient breathing cycles (approximately 10–30) for the calculation of our parameters.

Although the differences in respiratory motion characteristics between children and adults are smaller, our present results on intrafractional organ motion are in line with findings from our previous study, in which we demonstrated that interfractional abdominal organ motion in children differed from that in adults.^33^ This underscores the need, also in children, for a more individualized approach using 4DCT to define safety margins. However, all‐encompassing safety margins cannot be defined solely based on inter‐ and intrafractional motion. Setup variations and delineation errors,^6^ should also be taken into account, but to our knowledge have not been reported on for pediatric radiotherapy. Additionally, since respiratory‐induced diaphragm motion is used as a surrogate for abdominal organ or tumor motion, uncertainties need to be taken into account when these results are used for treatment planning purposes. Therefore, assumptions regarding potential margin reduction and dosimetric impact should be interpreted with caution. Nevertheless, as recently recommended by the Paediatric Radiation Oncology Society (PROS),^34^ consensus needs to be reached toward accurate margin definition in pediatric radiotherapy. With this study, we take another step closer toward developing guidelines for the appropriate approach to define accurate safety margins in pediatric radiotherapy.

## 5 Conclusions

In conclusion, these present results indicate that for children, a single pretreatment measurement of respiratory‐induced motion with 4DCT could be effective and could provide a good representation for intrafractional motion during radiotherapy. Moreover, large variation in amplitude and cycle time in both children and adults, confirms that 4DCT could be used for a more precise and individualized approach in pediatric radiotherapy, thereby aiming for more accurate safety margins and minimizing the risk of adverse events.

## Conflict of Interest

Dr. Alderliesten and Dr. Bel are project leaders of several Elekta‐sponsored projects outside of this work. Elekta had no involvement in study design, data collection and analyses, or writing of the manuscript.

## Supporting information


**Fig. S1.** Schematic overview of diaphragm motion tracking and respiratory‐induced diaphragm motion characteristics and analysis, acquired from [16].

**Fig. S2.** Scatter plots describing relations (Spearman's ρ and *P*‐value) between respiratory‐induced diaphragm motion characteristics and height and weight (significance level: *P *< 0.05).

**Table S1.** Pediatric patients characteristics.
**Table S2.** Adult patient characteristics.
