# Supplementary material for: The effectiveness of 4DCT in children and adults: A pooled analysis
Source: J Appl Clin Med Phys. 2018 Nov 9;20(1):276–83. doi: 10.1002/acm2.12488 (PMC6333119; doi:10.1002/acm2.12488)
Supplement: Supplementary file 1 — Fig. S1. Schematic overview of diaphragm motion tracking and respiratory‐induced diaphragm motion characteristics and analysis, acquired from [16]. [file ACM2-20-276-s001.docx]

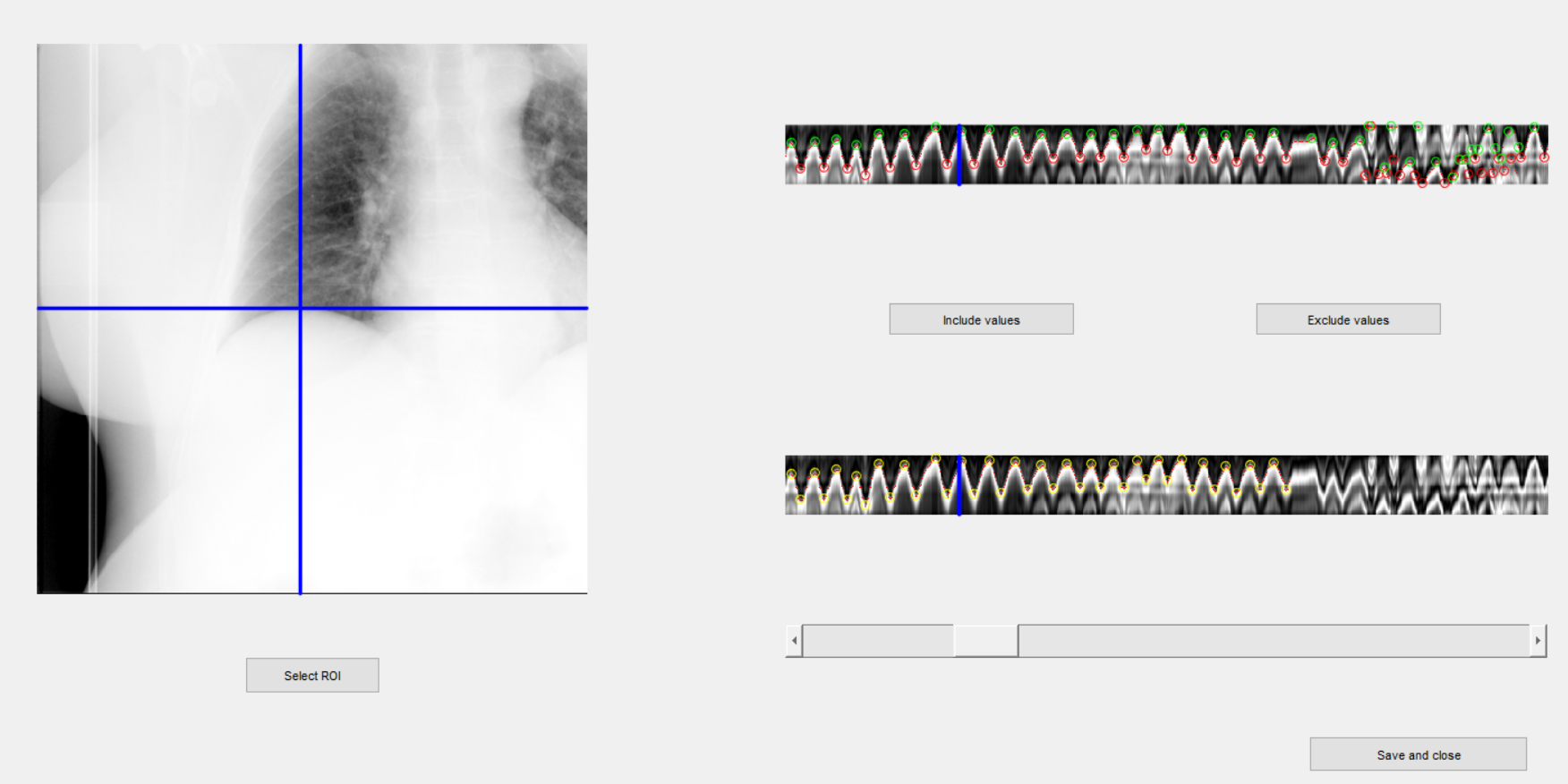

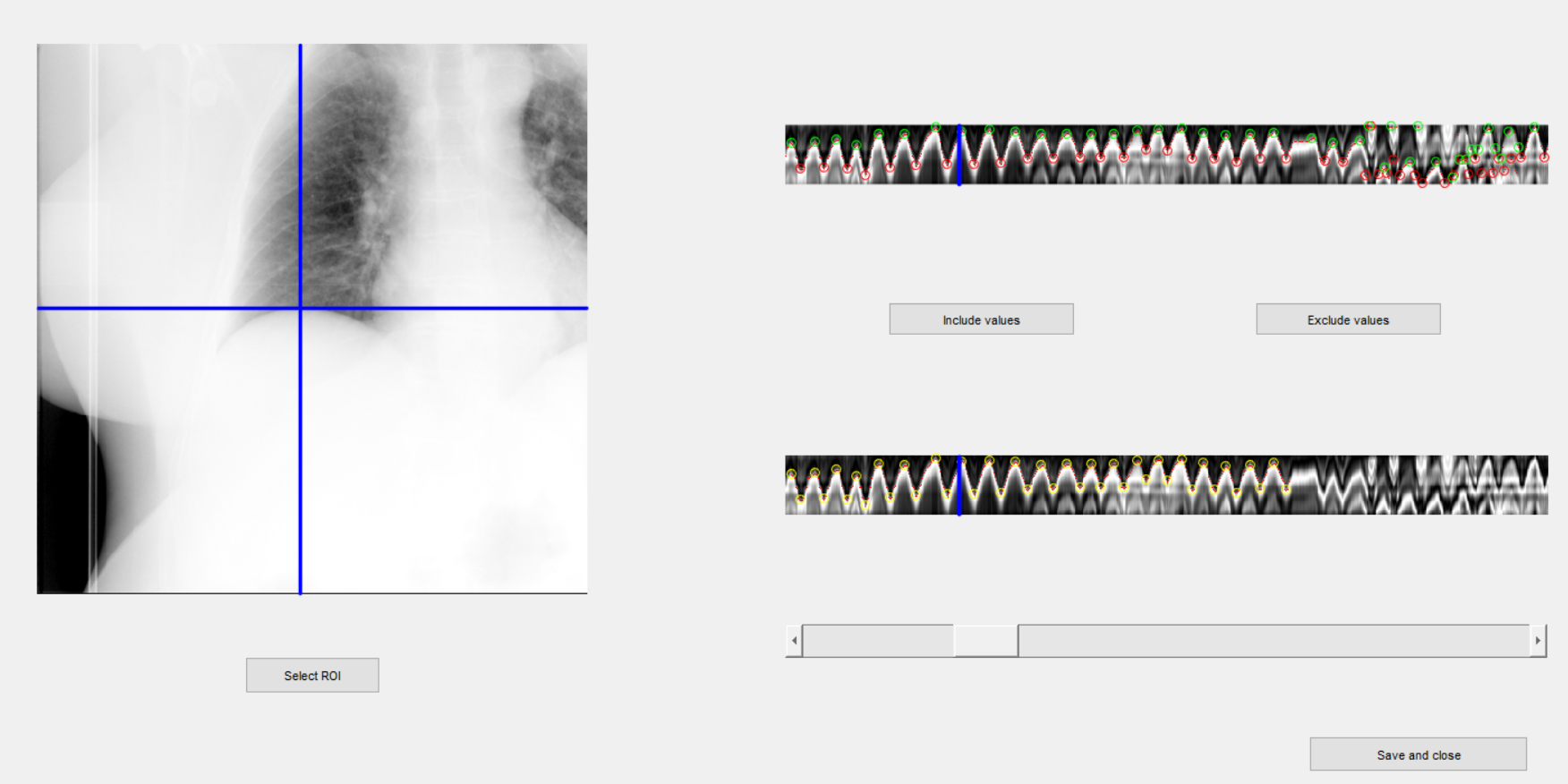

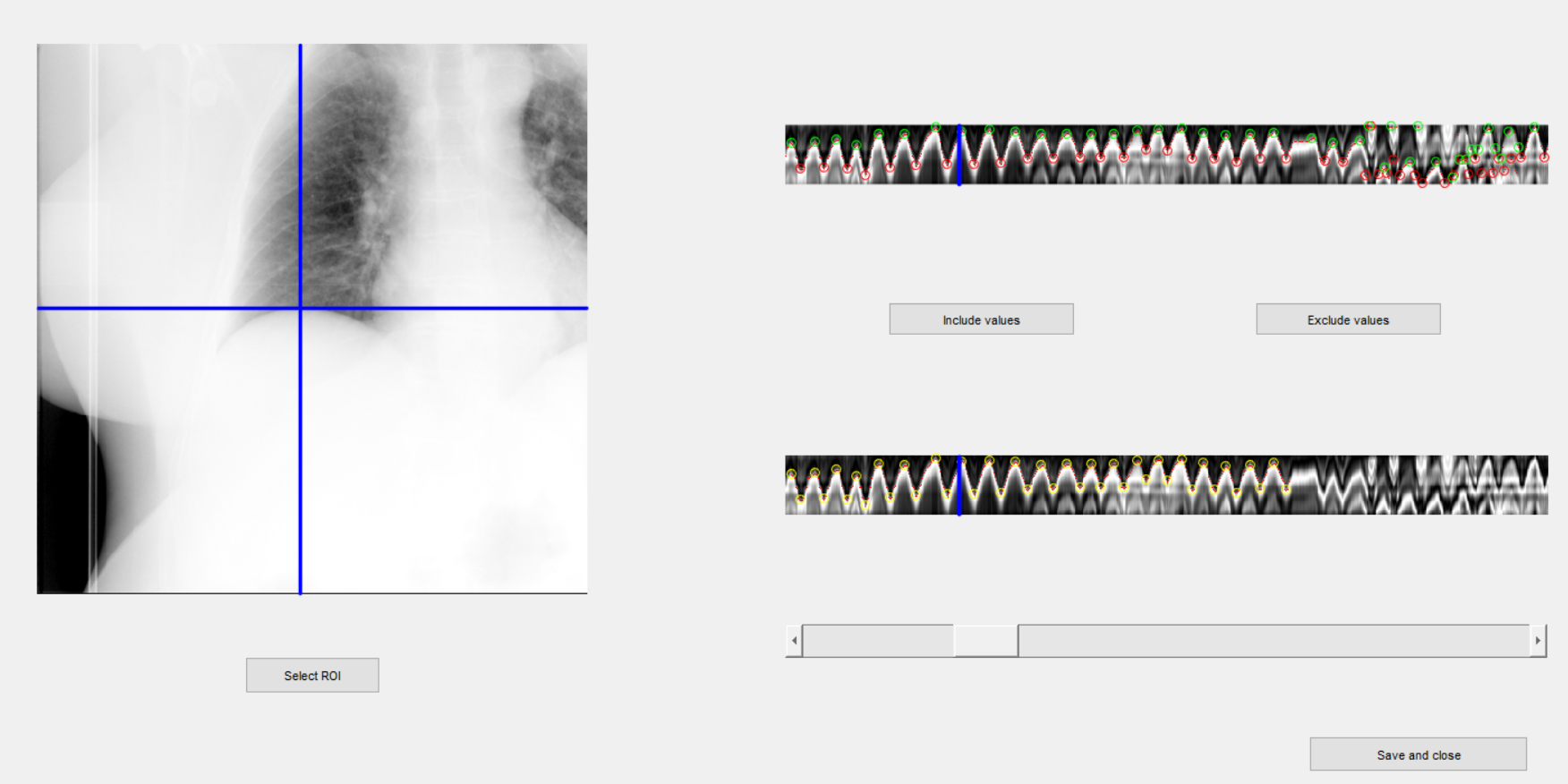

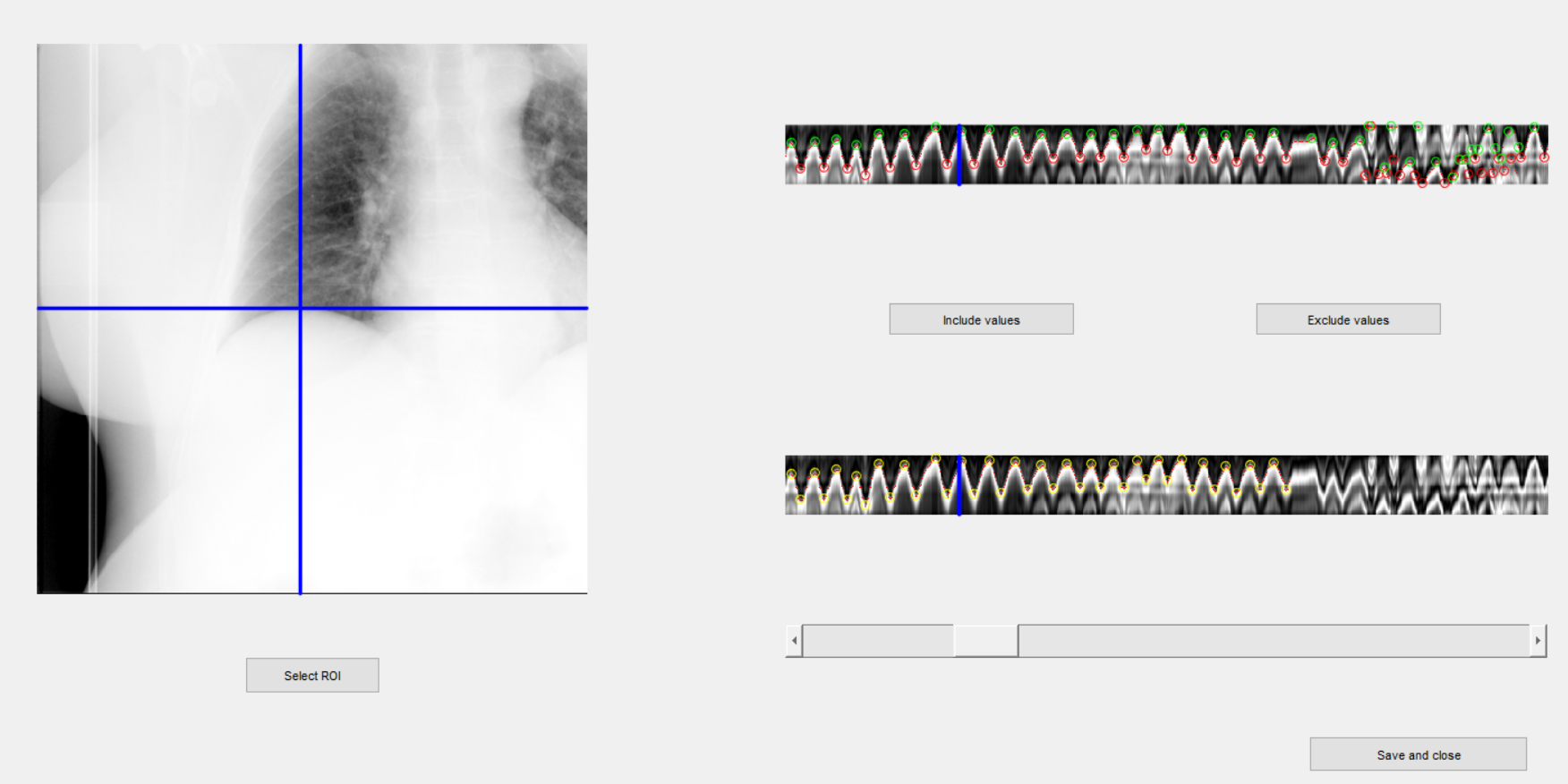

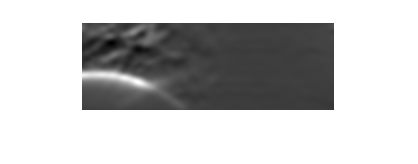

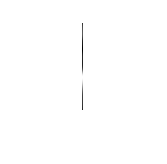

**Breathing Pattern**


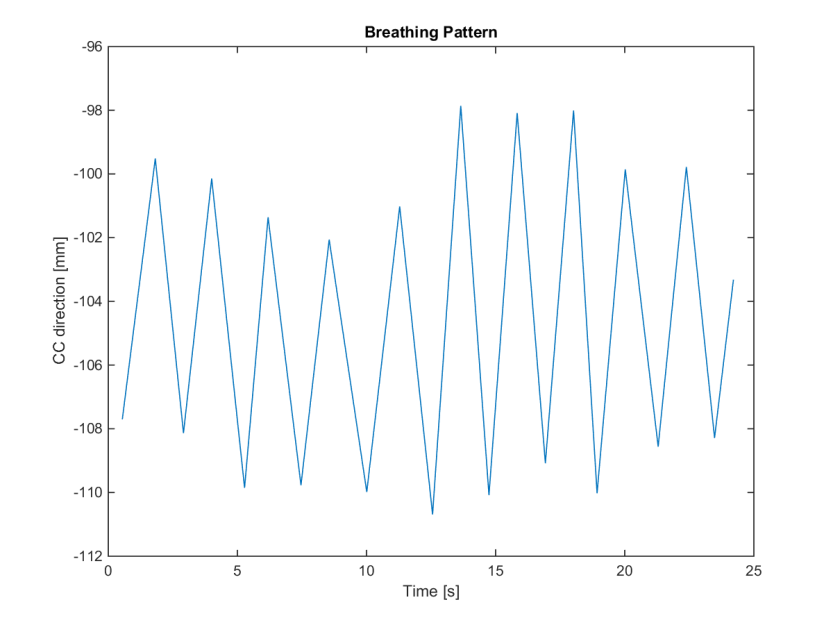


**Amplitude**

Per fraction:

- Mean amplitude and mean cycle time
- Intrafractional variability
  - SD over all amplitudes and cycle times

**CC direction (mm)**

**Time (s)**

**Cycle time**

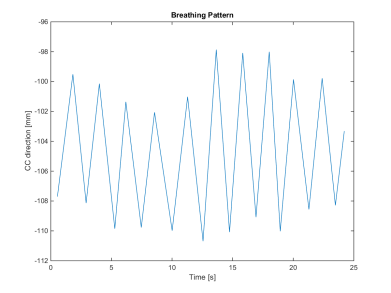

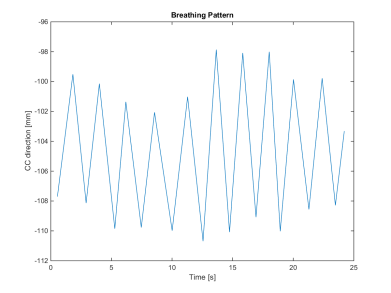

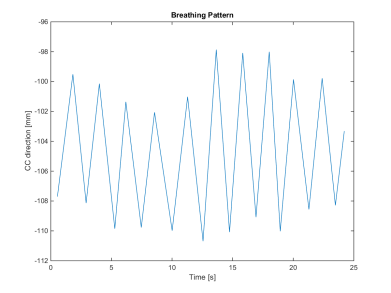


Per patient:

- Mean amplitude and mean cycle time
- Interfractional variability
  - SD over fraction means
- Intrafractional variability
  - root mean square of fraction SDs

**Supplementary Figure 1.** Schematic overview of diaphragm motion tracking and respiratory-induced diaphragm motion characteristics and analysis, acquired from [10].

1. A region of interest (ROI) was selected including the top of the right diaphragm (blue box).
2. The derivative of the grey values along the cranial-caudal (CC) direction (indicated by *v*) of the ROI was taken and the pixel values along each line (perpendicular to the CC direction, indicated by *u*) were summed, creating a one-dimensional image. This was repeated for all projection images.
3. Accumulating all one-dimensional images created a two-dimensional Amsterdam Shroud image. Subsequently, the pixel coordinates corresponding to the position of the diaphragm top were translated to millimeters relative to the patients’ planned isocenter and were also corrected for the geometry of the CBCT scanner, resulting in a respiratory signal as shown in d).
4. The amplitude was defined as the difference between the diaphragm position in the end-inhale and end-exhale phase. The cycle time equals inspiratory time plus expiratory time. Results were analysed per fraction,
5. per patient (over multiple fractions) and over the whole study population.
