# Supplementary material for: The effectiveness of 4DCT in children and adults: A pooled analysis
Source: J Appl Clin Med Phys. 2018 Nov 9;20(1):276–83. doi: 10.1002/acm2.12488 (PMC6333119; doi:10.1002/acm2.12488)
Supplement: Supplementary file 2 — Fig. S2. Scatter plots describing relations (Spearman's ρ and P‐value) between respiratory‐induced diaphragm motion characteristics and height and weight (significance level: P < 0.05). [file ACM2-20-276-s002.docx]

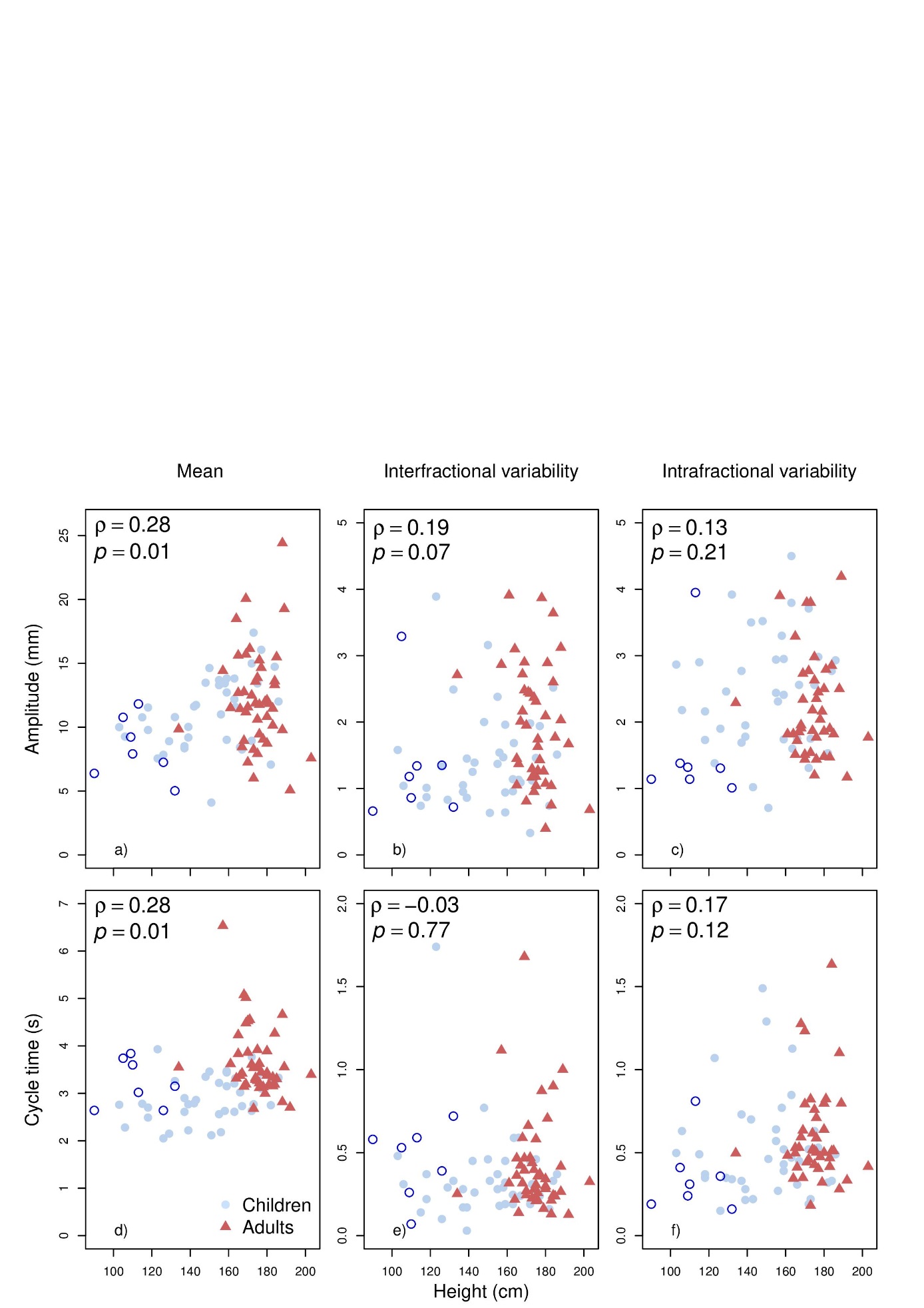


**Supplementary Figure 2A.** Scatter plots describing relations (Spearman’s ρ and *p*-value) between respiratory-induced diaphragm motion characteristics and height (significance level: *p<0.05*). Dots (light blue), open circles (dark blue) and triangles (red) represent respectively pediatric patients treated without and with anesthesia, and adult patients. (Color figure online only). Note: y-axes differ in range.


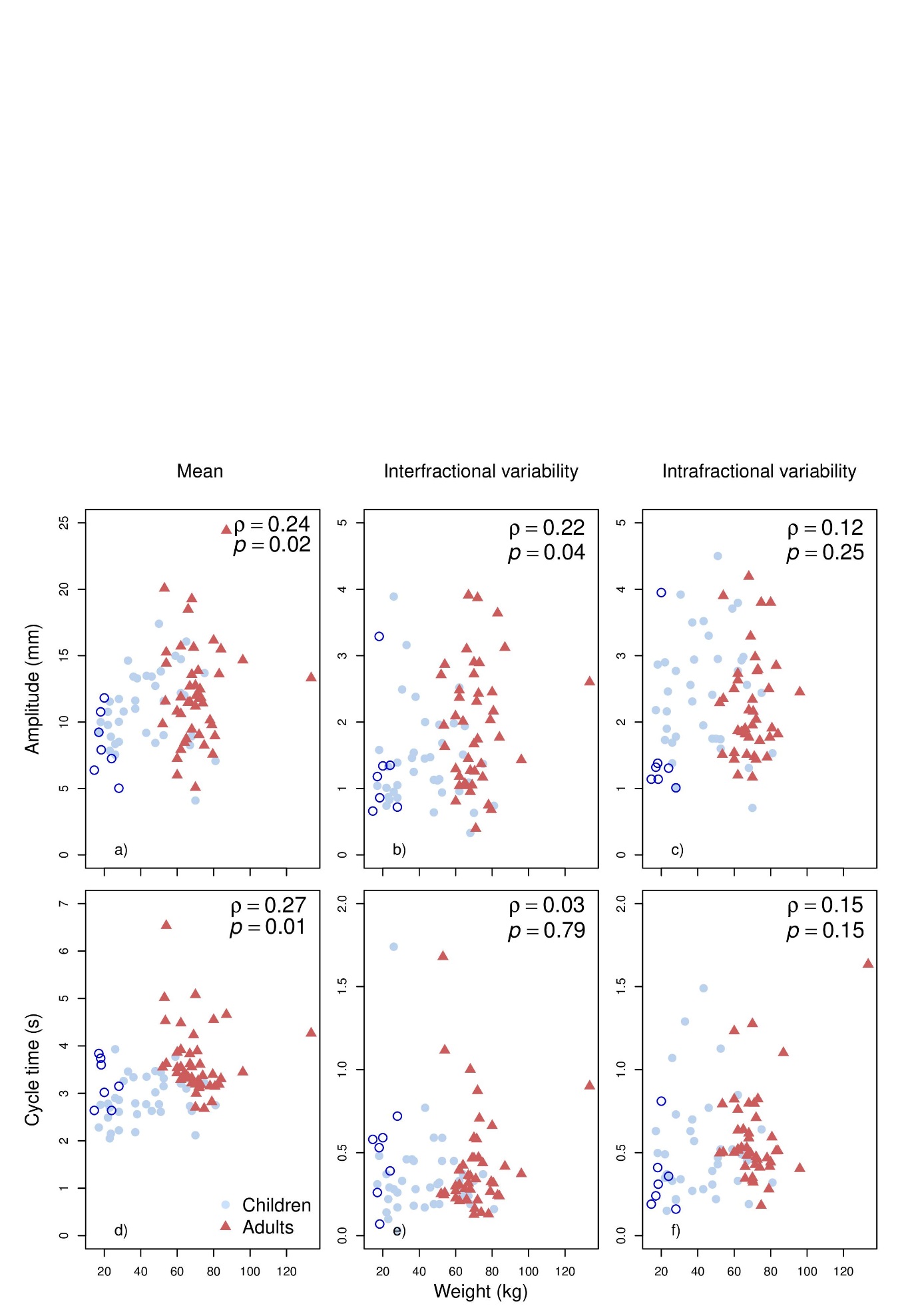


**Supplementary** **Figure 2B.** Scatter plots describing relations (Spearman’s ρ and *p*-value) between respiratory-induced diaphragm motion characteristics and weight (significance level: *p<0.05*). Dots (light blue), open circles (dark blue) and triangles (red) represent respectively pediatric patients treated without and with anesthesia, and adult patients. (Color figure online only). Note: y-axes differ in range.
