# Supplementary material for: The effectiveness of 4DCT in children and adults: A pooled analysis
Source: J Appl Clin Med Phys. 2018 Nov 9;20(1):276–83. doi: 10.1002/acm2.12488 (PMC6333119; doi:10.1002/acm2.12488)
Supplement: Supplementary file 3 — Table S1. Pediatric patients characteristics. Table S2. Adult patient characteristics. [file ACM2-20-276-s003.doc]

| **Supplementary Table 1.**  Pediatric patients characteristics and radiotherapy (RT) location. | | | | | | |  |  |
| --- | --- | --- | --- | --- | --- | --- | --- | --- |
| **No.** | **Gender** | **Tumour** | **Age at start RT (years)** | **Height (cm)** | **Weight (kg)** | **RT location** | **Number of CBCT** |  |
| 1 | M | Ewing sarcoma | 10.9 | 137 | 28 | Thorax | 6 |  |
| 2 | F | Ewing sarcoma | 11.6 | 155 | 38 | Thorax | 27 |  |
| 3 | M | Medulloblastoma | 11.0 | 158 | 46 | CSI | 4 |  |
| 4 | M | Medulloblastoma | 10.9 | 150 | 33 | CSI | 5 |  |
| 5 | F | Ewing sarcoma | 17.9 | 164 | 53 | Thorax | 17 |  |
| 6^a^ | M | Medulloblastoma | 6.6 | 110 | 18 | CSI | 5 |  |
| 7 | F | Ewing sarcoma | 12.5 | 151 | 70 | Thorax | 32 |  |
| 8 | M | Medulloblastoma | 14.1 | 175 | 36 | CSI | 9 |  |
| 9 | F | Medulloblastoma | 8.2 | 139 | 28 | CSI | 7 |  |
| 10 | F | Neuroblastoma | 6.2 | 103 | 18 | Thorax | 23 |  |
|  |  |  |  |  |  |  |  |  |
| 11 | M | CCSK | 8.6 | 126 | 23 | Abdomen | 5 |  |
| 12 | M | Ewing sarcoma | 16.8 | 184 | 62 | Thorax | 8 |  |
| 13^a,b^ | M | Medulloblastoma | 7.6 | 126 | 24 | CSI | 6 |  |
| 14 | F | Germinoma | 9.3 | 139 | 43 | CSI | 6 |  |
| 15 | M | Medulloblastoma | 6.7 | 129 | 24 | CSI | 5 |  |
| 16 | F | Hodgkin lymphoma | 15.9 | 163 | 51 | Thorax | 6 |  |
| 17 | F | Medulloblastoma | 7.0 | 118 | 22 | CSI | 7 |  |
| 18 | M | Medulloblastoma | 11.0 | 143 | 28 | CSI | 4 |  |
| 19^b^ | M | Pineal germinoma | 15.8 | 159 | 48 | CSI | 17 |  |
| 20 | M | Pineal germinoma | 13.3 | 166 | 48 | CSI | 6 |  |
| 21 | M | Medulloblastoma | 6.2 | 123 | 23 | CSI | 10 |  |
| 22 | M | Ewing sarcoma | 14.7 | 177 | 65 | Thorax | 10 |  |
| 23 | F | Ewing sarcoma | 15.8 | 173 | 50 | Thorax | 8 |  |
| 24  25^a^ | F  F | B-cell lymphoma  Ependymoma | 16.8  5.6 | 163  113 | 62  20 | Thorax  Spinal | 12 |  |
|  |  |  |  |  |  |  | 21 |  |
| 26 | F | Ewing sarcoma | 13.1 | 148 | 43 | Thorax | 10 |  |
| 27 | M | ERMS | 16.8 | 186 | 64 | Thorax | 5 |  |
| 28 | M | Glioma | 7.8 | 132 | 31 | CSI | 14 |  |
| 29 | F | Medulloblastoma | 14.7 | 159 | 50 | CSI | 9 |  |
| 30^a^ | M | Medulloblastoma | 5.1 | 109 | 17 | CSI | 8 |  |
| 31 | F | Neuroblastoma | 5.3 | 115 | 24 | Abdomen | 7 |  |
| 32 | F | Medulloblastoma | 12.5 | 156 | 37 | CSI | 6 |  |
| 33 | M | RMS | 10.9 | 142 | 37 | Thorax | 25 |  |
| 34 | M | Wilms’ tumour | 15.6 | 167 | 67 | Abdomen | 7 |  |
| 35 | M | Germinoma | 16.9 | 172 | 68 | CSI | 6 |  |
| 36 | M | DSRCT | 9.9 | 137 | 26 | Abdomen | 5 |  |
| 37 | M | ERMS | 3.3 | 106 | 17 | Abdomen | 24 |  |
| 38 | M | Neuroblastoma | 4.7 | 118 | 22 | Thorax | 6 |  |
| 39 | F | Ewing sarcoma | 17.4 | 154 | 78 | Thorax | 16 |  |
| 40^a^ | M | Medulloblastoma | 4.9 | 105 | 18 | CSI | 10 |  |
| 41 | M | Ewing sarcoma | 17.8 | 182 | 81 | Thorax | 28 |  |
| 42 | F | Osteosarcoma | 15.1 | 159 | 53 | Thorax | 6 |  |
| 43^a^ | M | ERMS | 10.7 | 132 | 28 | Abdomen | 4 |  |
| 44^a^ | M | Neuroblastoma | 2.2 | 90 | 15 | Abdomen | 6 |  |
| 45 | M | RMS | 14.4 | 172 | 59 | Thorax | 12 |  |
| *Abbreviations:* CBCT = cone beam computed tomography; M = male; F = female; CCSK = Clear Cell Sarcoma Kidney; (E)RMS = (Embryonal) Rhabdomyosarcoma; DSRCT = Desmoplastic small round cell tumour; CSI = Craniospinal Irradiation  ^a^ Patients were treated under general anaesthesia (GA)  ^b^ Patients were treated in prone position | | | | | | |  |  |

| **Supplementary Table 2.** | | | | | | |
| --- | --- | --- | --- | --- | --- | --- |
| Adult patient characteristics | | | | | | |
| **No.** | **Gender** | **Tumour** | **Age at start RT (years)** | **Height (cm)** | **Weight (kg)** | **Number of CBCT** |
| 1 | F | Gastric carcinoma | 70 | 169 | 53 | 12 |
| 2 | M | Pancreatic cancer | 53 | 203 | 80 | 2 |
| 3 | F | Oesaphagus carcinoma | 69 | 169 | 62 | 7 |
| 4 | F | Gastric carcinoma | 45 | 175 | 62 | 7 |
| 5 | M | Pancreatic cancer | 69 | 183 | 66 | 15 |
| 6 | M | Gastric carcinoma | 63 | 175 | 72 | 25 |
| 7 | F | Pancreatic cancer | 52 | 165 | 67 | 15 |
| 8 | M | Pancreatic cancer | 71 | 180 | 60 | 11 |
| 9 | M | Pancreatic cancer | 70 | 180 | 65 | 11 |
| 10 | F | Pancreatic cancer | 74 | 157 | 54 | 15 |
| 11 | F | Oesaphagus carcinoma | 68 | 164 | 66 | 8 |
| 12 | M | Pancreatic cancer | 68 | 175 | 62 | 11 |
| 13 | M | Oesaphagus carcinoma | 44 | 184 | 134 | 12 |
| 14 | M | Gastric carcinoma | 52 | 173 | 75 | 6 |
| 15 | M | Oesaphagus carcinoma | 66 | 180 | 71 | 2 |
| 16 | M | Gastric carcinoma | 66 | 189 | 68 | 11 |
| 17 | M | Pancreatic cancer | 60 | 174 | 62 | 25 |
| 18 | M | Oesaphagus carcinoma | 93 | 168 | 81 | 14 |
| 19 | F | Gastric carcinoma | 55 | 176 | 54 | 8 |
| 20 | M | Pancreatic cancer | 66 | 171 | 80 | 15 |
| 21 | M | Oesaphagus carcinoma | 65 | 188 | 87 | 12 |
| 22 | M | Gastric carcinoma | 56 | 188 | 79 | 25 |
| 23 | M | Oesaphagus carcinoma | 42 | 169 | 70 | 5 |
| 24 | M | Gastric carcinoma | 52 | 176 | 68 | 6 |
| 25 | M | Pancreatic cancer | 42 | 183 | 78 | 15 |
| 26 | M | Gastric carcinoma | 62 | 176 | 72 | 6 |
| 27 | M | Gastric carcinoma | 52 | 166 | 74 | 6 |
| 28 | M | Gastric carcinoma | 68 | 173 | 60 | 5 |
| 29 | F | Gastric carcinoma | 41 | 161 | 67 | 6 |
| 30 | F | Oesaphagus carcinoma | 34 | 177 | 96 | 7 |
| 31 | M | Gastric carcinoma | 43 | 185 | 84 | 5 |
| 32 | M | Oesaphagus carcinoma | 70 | 172 | 73 | 10 |
| 33 | M | Oesaphagus carcinoma | 46 | 174 | 68 | 9 |
| 34 | F | Pancreatic cancer | 77 | 167 | 64 | 23 |
| 35 | M | Oesaphagus carcinoma | 67 | 178 | 72 | 9 |
| 36 | F | Pancreatic cancer | 67 | 134 | 52 | 12 |
| 37 | F | Pancreatic cancer | 58 | 170 | 60 | 14 |
| 38 | M | Gastric carcinoma | 61 | 179 | 70 | 6 |
| 39 | M | Gastric carcinoma | 62 | 171 | 69 | 25 |
| 40 | M | Pancreatic cancer | 78 | 184 | 83 | 30 |
| 41 | M | Pancreatic cancer | 80 | 181 | 73 | 11 |
| 42 | M | Gastric carcinoma | 51 | 192 | 70 | 7 |
| 43 | F | Oesaphagus carcinoma | 46 | 168 | 70 | 24 |
| 44 | F | Oesaphagus carcinoma | 75 | 165 | 69 | 7 |
| 45 | M | Gastric carcinoma | 68 | 170 | 54 | 7 |
| *Abbreviations:* CBCT = cone beam computed tomography; M = male; F = female, RT = radiotherapy | | | | | | |
